# Antenatal ultrasound diagnosis of small bowel non-rotation in complex left isomerism: a case report

**DOI:** 10.1016/j.ijscr.2018.11.069

**Published:** 2019-02-19

**Authors:** Charles Arcus, Usha Sennaiyan, Amit Trivedi, Thushari I. Alahakoon

**Affiliations:** a Westmead Institute for Maternal and Fetal Medicine, Westmead Hospital, Sydney, NSW, 2145, Australia; b The University of Sydney, Sydney Medical School, Sydney, NSW, Australia; c Grace Centre for Newborn Care, Westmead Children’s Hospital, Sydney, NSW, 2145, Australia

**Keywords:** Heterotaxy, Isomerism, Non-rotation, Laterality, Antenatal diagnosis, Case report

## Abstract

**Presentation of case:**

A multiparous expectant mother was referred to our tertiary unit at 23 weeks with a complex fetal cardiac anomaly in the context of suspected heterotaxy syndrome. The cardiac findings were consistent with isomerism: the fetal cardiac position was levocardia with a single functioning double outlet ventricle and AV valve, pulmonary stenosis, and interrupted inferior vena cava (IVC) with azygous continuation. The fetal abdominal situs was also altered, with the stomach to the right, and the hepatobiliary system midline to left. The spleen was not identified antenatally or postnatally. At 36 weeks, ultrasound revealed an abnormal bowel pattern with small bowel loops on the right side of the abdomen and large bowel on the left, suggesting a diagnosis of non- rotation. The infant was delivered vaginally at 39 weeks. The cardiac diagnosis and non-rotation of the small bowel were confirmed by postnatal echocardiography and contrast fluoroscopy.

**Discussion:**

Heterotaxy syndrome is traditionally classified into right or left isomerism depending on how and where the organs are anatomically arranged. The case presented here demonstrates mixed laterality and prenatal ultrasound features of non-rotation.

**Conclusion:**

It is important to be informed of the embryological variants of isomerism and actively seek antenatal evidence of bowel non-rotation in such cases.

## Highlights

• A rare case of mixed isomerism and antenatally diagnosed non-rotation of bowel is reported.
• Features of bowel non-rotation should be sought antenatally in cases of isomerism.
• Mixed isomerism and postnatal sequelae should be considered when right and left sided pathology coexist.z

## 1 Introduction

Heterotaxy syndrome or isomerism refers to the abnormal arrangement of thoracoabdominal organs. In embryological development, the left/right axis of the body normally rotates but in the setting of isomerism, the axis is disrupted, and the organs are not laid out asymmetrically. There are many known cardiac and extra-cardiac complications associated with this condition [1] but the antenatal diagnosis of bowel non-rotation in the context of heterotaxy has not been widely reported in the literature. We present a case of a multiparous expectant mother who was referred to our unit with suspected isomerism with mixed laterality where non-rotation of the fetal bowel was diagnosed on antenatal ultrasound. Our work is reported in line with the SCARE criteria [2].

## 2 Presentation of case

A 33-year-old healthy multipara with no notable genetic, drug, psychosocial, or family history was referred to our tertiary unit at 23 weeks following a second trimester morphology scan with a complex cardiac abnormality in the context of suspected isomerism. She had two term vaginal deliveries of healthy infants and her medical history included seasonal asthma and uterine fibroids. The current pregnancy was spontaneously conceived, and her antenatal history and the results of first trimester ultrasound and chromosomal screening were low risk.

**Fig. 1:**
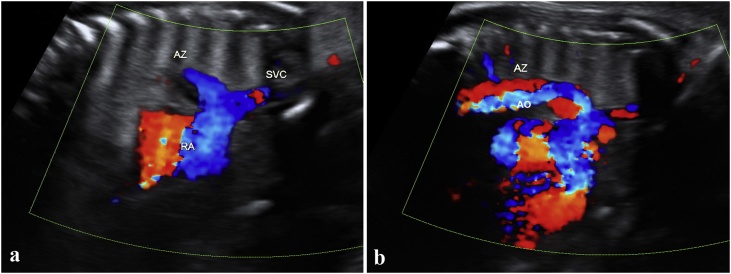
Vascular connections to the heart a: Confluence of azygous vein (AV) and superior vena cava (SVC) entering the right atrium b: Right sided descending aorta and the azygous vein. RA = Right atrium.

A scan performed at our Maternal Fetal Medicine (MFM) Unit by trained departmental ultrasonagraphers revealed fetal growth was within low normal range and the estimated fetal weight on the 15th percentile. At 26 weeks, the stomach was visualized on the right but neither the spleen or the gall bladder was identifiable. The small bowel pattern appeared unremarkable. The fetal heart position was levocardia. The outflow tracts were noted to be parallel and arising from the single left ventricle. A diagnosis of single ventricle, single atrioventricular connection, and double outlet ventricle with mild to moderate pulmonary stenosis was made. The IVC appeared interrupted with azygous vein continuation (Fig. 1a) to the right sided superior vena cava (SVC). The aortic arch was right sided with uninterrupted flow (Fig. 1b). Two atrial chambers were noted, and the pulmonary veins seemed to drain into the posterior right sided atrium. There were no signs of heart block or hydrops. A fetal echocardiogram by a pediatric cardiologist confirmed the findings. Although a diagnosis of left heterotaxy syndrome was made, the fetus displayed features of both left and right isomerism.

**Fig. 2:**
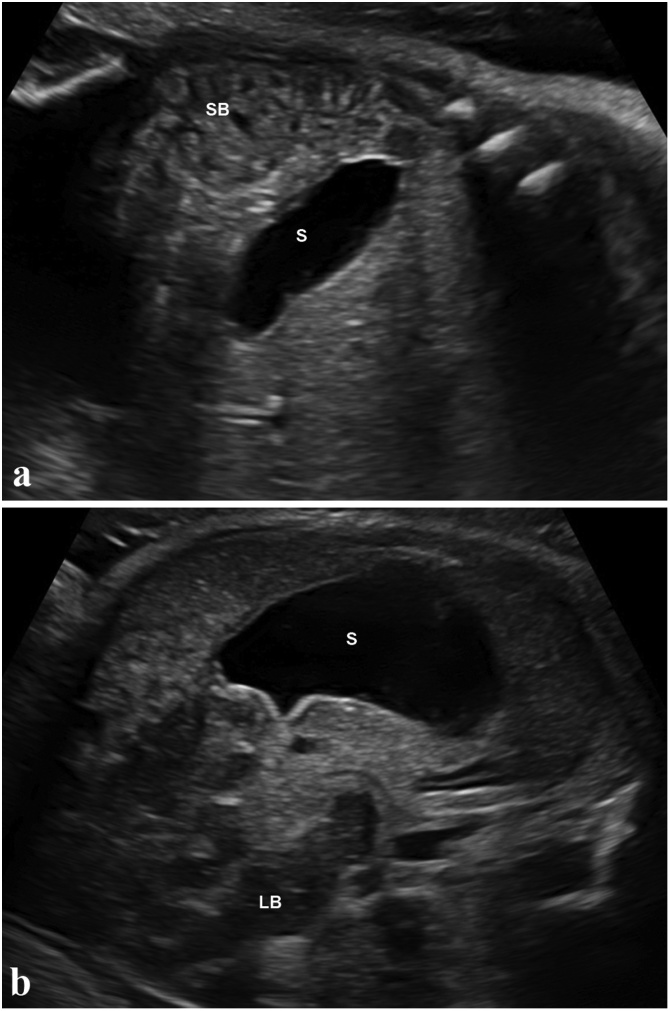
Transverse sections of the fetal abdomen showing a: fetal stomach (S) and small bowel (SB) on the right side of the abdomen b: fetal large bowel (LB) on the left side of the abdomen.

**Fig. 3:**
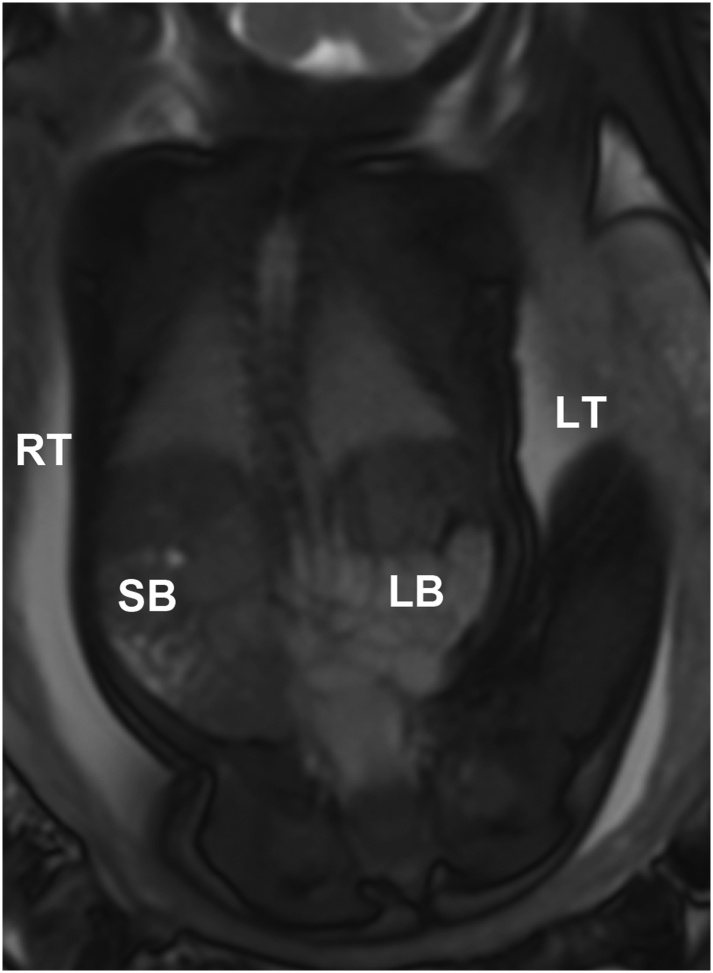
MRI imaging of 39 week fetus showing the bowel non-rotation in a coronal plane. SB = small bowel. LB = large bowel. LT = left side of the fetus. RT = right side of the fetus.

A repeat ultrasound at 32 weeks noted small bowel loops to be on the right side of the fetal abdomen and the large bowel loops were arranged to the left (Fig. 2 a and b). There was no evidence of bowel dilatation or ascites and the bowel wall thickness was within normal limits. The superior mesenteric artery was noted to on the right side of the aorta, which was consistent with an altered superior mesenteric artery/superior mesenteric vein axis and suggested non-rotation [3]. During follow-up scans at 36 and 39 weeks, non-rotation of the bowel was reaffirmed with no evidence of bowel dilatation or obstruction. The antenatal diagnosis of non-rotation in the context of heterotaxy syndrome was made and pediatric surgical referral was suggested. A fetal MRI performed at 39 weeks also suggested absent spleen, gall bladder and confirmed non-rotation (Fig. 3). However, the MRI did not provide any additional information about the lung and bronchial morphology due to the advanced gestational age. Hence, the laterality of isomerism remained inconclusive.

**Fig. 4:**
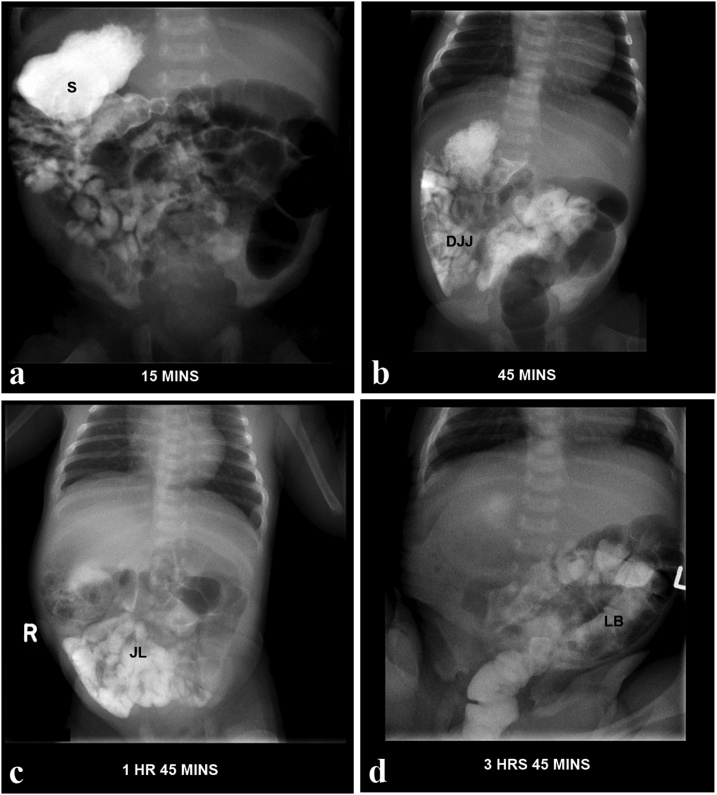
Barium study of the neonatal bowel a: the stomach (S) within the right upper abdomen b: the duodenal-jejunal junction (DJJ) located right of the midline c: the jejunal loops (JL) predominantly right sided d: the sigmoid colon (SC) and opacified large bowel on the left.

The infant was delivered at 39 weeks by uncomplicated vaginal birth and weighed 3.7 kg. APGARS were 9 and 9 at one, and five minutes respectively. Postnatally, the cardiac and extra-cardiac findings of heterotaxy syndrome were confirmed. Postnatal echocardiogram confirmed probable left atrial isomerism, IVC interruption with azygous continuation, a single indeterminate ventricle, a VSD, sub pulmonary stenosis, pulmonary valve stenosis and a persistent ductus arteriosus. On the postnatal abdominal ultrasound, the liver was seen midline, but the spleen and gall bladder were absent. Postnatal barium study results showed the stomach within the right upper abdomen (Fig. 4a), the duodenal-jejunal junction located right of the midline (Fig. 4b), the jejunal loops predominantly right sided (Fig. 4c) and the sigmoid colon and opacified large bowel on the left (Fig. 3d).

Clinically, the infant fed well and bowels were opened. Abdominal examination and XR radiograph were unremarkable, and so any concerns of volvulus and need for intra-abdominal operative intervention were allayed. Amoxycillin was given for asplenic prophylaxis and she was discharged home with follow up arranged with cardiology and surgical teams respectively.

At 2 months of age prior to corrective cardiac surgery, a CT was also performed and showed features consistent with complex left heterotaxy/isomerism with azygous continuation of the inferior vena cava, bilobed lungs, left-sided liver and intestinal non- rotation. There was a dominant morphologic left ventricular cardiac chamber with a large VSD and a rudimentary right ventricle.

## 3 Discussion

The strengths of the case presented are that it highlights two important aspects of heterotaxy that may be relevant in managing these cases: 1) heterotaxy cannot always be classified into right or left isomerism alone and 2) non-rotation of the bowel can be diagnosed antenatally.

### 3.1 Laterality

In isomerism or heterotaxy syndrome, visceral asymmetry is lost as the left/right axis of the body fails to rotate embryologically. Traditionally, the condition is classified into left and right isomerism. The former is also called polysplenia, a “laterality disturbance associated with paired left sided viscera” [4] associated with cardiac anomalies (including conduction anomalies [5] and bilateral left atrial appendages), and extra- cardiac malformations (including bilobed lungs, long hyper arterial bronchi and a right sided stomach) [6]. An important anatomical marker of left isomerism is interruption of the IVC, with azygous continuation [7]. If this is present, one should have a high index of suspicion for left isomerism. Common extra-cardiac abnormalities of left isomerism are biliary atresia, gut mal-rotation or non-rotation [8], and potential volvulus [9]. Although serious, these extra-cardiac anomalies afford less morbidity than the congenital cardiac defects [10] that can be associated too, albeit in as low as 3% of cases [11].

The cardiac defects in right atrial isomerism are different. They may include a common atrioventricular canal, a doublet outlet ventricle, transposition of the great arteries, and pulmonary stenosis [12]. Right isomerism is also characterized by two trilobed lungs and asplenia. Interruption of the IVC with azygous continuation is not, however, a feature of right isomerism and a useful differentiating marker between the two subcategories.

In the case presented, laterality was mixed. There were features of right isomerism (asplenia, single ventricular physiology, and abnormal pulmonary venous drainage) and left isomerism (IVC interruption with azygous continuation and gut non-rotation). In cases such as these where traditional dichotomies used to describe the condition do not apply, it is important we acknowledge a mixed picture and use postnatal imaging to guide diagnosis and anticipate postnatal complications.

### 3.2 Gut non-rotation

Gut mal-rotation or non-rotation are complications commonly associated with heterotaxy syndromes. Children with heterotaxy have a gut rotational anomaly in as many as 40–90% of cases [13]. In one study, 10 of 27 infants with isomerism had gut mal-rotation and 2 developed volvulus [14]. In other studies, the incidence of mal- rotation in isomerism is quoted as high as 60%–70% [15,16] whilst another paper argues isomerism confers a 52-fold increased risk of mal-rotation but not volvulus [17]. Although many concede that “there is great variation in how heterotaxy associated defects are diagnosed, described and reported” [18], it is uncontested that bowel mal-rotation is a significant clinical sequelae.

Historically, the diagnosis of rotational intestinal anomaly in isomerism is made when infants present with billous vomiting or feeding intolerance that lead to gastrointestinal imaging and surgical referral [19]. The operation required is a Ladd’s Procedure whereby peritoneal bands are divided and the mesenteric stalk of the small bowel widened, to detort the bowel, and reduce the risk of bowel ischaemia [20].

The operation carries risk [21] because the cohort of patients who require it are medically complex. Concurrent cardiac anomalies pose substantial perioperative morbidity [22,23] and the mortality rate is reported as high as 15–21% [[24], [25], [26]].

Currently, there is no consensus on whether or not we should perform an elective Ladd’s for such patients at all. Many argue that it is ill-advised given the risk of volvulus is so low [[27], [28], [29]] and that it is safest to surgically intervene in symptomatic patients alone [30], especially given the procedure does not always alleviate partial or complete obstruction [31]. Others argue, however, that the operation may be justified in the asymptomatic patient if the mesenteric base is thought to be narrow [32,33] and the of risk of volvulus increased.

If gut non-rotation in the setting of isomerism is diagnosed antenatally as it is in the case presented, then care-providers can refer to subspecialist services at an earlier junction and make a more considered assessment of the need for surgery.

## 4 Conclusion

We present a rare case of mixed isomerism with antenatal features of bowel non- rotation. Antenatal diagnosis by way of ultrasound of such a rare but significant condition may be limited by operator dependence but is well tolerated by the patient and enables medical professionals to make an earlier referral to subspecialist services, and better counsel patients of what may be expected. Highlighting a case of mixed isomerism also challenges traditional dichotomies used to define heterotaxy syndrome and encourages medical professionals to anticipate sequelae of both right and left isomerism simultaneously.

## Conflicts of interest

I do not have any conflicts of interest.

## Funding

There is no source of funding for this paper.

## Ethical approval

The case report has been ethically approved by The Executive of the Sydney Children's Hospitals Network Human Research Ethics Committee (HREC).

## Consent

Written informed consent was obtained from the patient for publication of this case report and accompanying images. A copy of the written consent is available for review by the Editor- in-Chief of this journal on request.

## Author contribution

Dr Charles Arcus is the primary author and wrote the paper.

Dr Usha Sennaiyan was involved in editing the paper.

Dr Amit Trivedi was involved in data collection and editing.

Dr Indika Alahakoon was involved in editing, data collection, data interpretation, and the study design.

## Registration of research studies

Not applicable to this study.

## Guarantor

Dr Charles Arcus and Dr Indika Alahakoon.

## Provenance and peer review

Not commissioned, externally peer-reviewed.
